# Premedication with midazolam in intellectually disabled dental patients: Intramuscular or oral administration? A retrospective study

**DOI:** 10.4317/medoral.21086

**Published:** 2016-03-31

**Authors:** Hiroshi Hanamoto, Aiji Boku, Mitsutaka Sugimura, Aiko Oyamaguchi, Mika Inoue, Hitoshi Niwa

**Affiliations:** 1 Department of Dental Anesthesiology, Osaka University Graduate School of Dentistry

## Abstract

**Background:**

The use of midazolam for dental care in patients with intellectual disability is poorly documented. The purpose of this study was to determine which method of premedication is more effective for these patients, 0.15 mg/kg of intramuscular midazolam or 0.3 mg/kg of oral midazolam.

**Material and Methods:**

This study was designed and implemented as a non-randomized retrospective study. The study population was composed of patients with intellectual disability who required dental treatment under ambulatory general anesthesia from August 2009 through April 2013. Patients were administered 0.15 mg/kg of midazolam intramuscularly (Group IM) or 0.3 mg/kg orally (Group PO). The predictor variable was the method of midazolam administration. The outcome variables measured were Observer’s Assessment of Alertness/ Sedation (OAA/S) Scale scores, the level of cooperation when entering the operation room and for venous cannulation, post-anesthetic agitation and recovery time.

**Results:**

Midazolam was administered intramuscularly in 23 patients and orally in 21 patients. More patients were successfully sedated with no resistance behavior during venous cannulation in Group PO than in Group IM (*p*=0.034). There were no differences in demographic data and other variables between the groups.

**Conclusions:**

The results of this study suggest that oral premedication with 0.3 mg/kg of midazolam is more effective than 0.15 mg/kg of midazolam administered intramuscularly, in terms of patient resistance to venous cannulation. If both oral and intramuscular routes of midazolam are acceptable in intellectually disabled patients, the oral route is recommended.

**Key words:**Premedication, midazolam, intellectual disability.

## Introduction

The number of people with special needs who require oral health services is increasing (1). Patients with special needs, such as those with intellectual disability, are sometimes uncooperative for medical procedures, especially dental procedures. For such patients, general anesthesia is a useful method that enables completion of dental treatment. When general anesthesia is applied to intellectually disabled dental patients, sedation by premedication is essential to reduce induction anxiety. Since general anesthesia for such patients is usually given in an ambulatory setting in which rapid recovery is required, the route of administration of the premedicant is important.

Although several drugs, such as ketamine (2), clonidine (3) and dexmedetomidine (4,5), are used as sedative premedication in pediatric patients, midazolam is one of the most popular sedatives. However, little is known about the ideal dose or route of administration of midazolam for intellectually disabled patients undergoing ambulatory surgery. We performed this study to compare the effects of oral versus intramuscular midazolam as premedication before dental surgery under general anesthesia in intellectually disabled subjects. This study was designed as a non-randomized retrospective study because most patients and their parents or caregivers preferred the oral route for administration of the premedicant, and hence, the patients could not be randomly allocated to the two groups.

## Material and Methods

To address the research purpose, the investigators designed and implemented a retrospective study to evaluate the effectiveness of intramuscular and oral administration of midazolam as sedative premedication before general anesthesia in intellectually disabled patients. This study was performed according to the Declaration of Helsinki and approved by the institutional review board and ethics committee of Osaka University Dental Hospital (H26-E50).

- Patients

The study population was composed of patients with intellectual disability receiving dental treatment under ambulatory general anesthesia from August 2009 through April 2013. To be included in the study, patients had to be American Society of Anesthesiologists Physical Status (ASA-PS) class I or II, and aged over 16 years old. Patients were excluded from participation if their body weight was over 67 kg, because the oral dose of midazolam was limited to a maximum of 20 mg in this study. Patients who could not walk normally, declined measurement of their BP, or whose perioperative records were incomplete, were also excluded. All study variables were collected from the medical or anesthesia records.

- Standard ambulatory anesthesia procedure

Patients were admitted to the hospital by 8:30 a.m. after more than 8 hours of fasting. If patients regularly took a morning dose of tranquillizers or anti-epilepsy medication, they were asked to take these drugs between 6:00 and 7:00 a.m. with a little plain water. After entering the day surgery preparation room, patients were administered midazolam (Dormicum, Astellas, Tokyo, Japan) intramuscularly (0.15 mg/kg: Group IM) or orally (0.3 mg/kg: Group PO) as premedication. Orally administered midazolam was limited to a maximum dose of 20 mg (6). The oral premedication syrup was prepared by mixing midazolam in a sucrose based syrup (Simple syrup, Mylan, Tokyo, Japan). The administration route was determined according to the parent’s request and patient’s acceptance on the day of preoperative examination.

Patients’ sedation level was evaluated by the dental anesthesiologist approximately 15 and 30 min after premedication in Group IM and Group PO, respectively, using the Observer’s Assessment of Alertness/Sedation (OAA/S) scale (7). OAA/S scale scores are rated as follows: 5, awake and responds readily to name spoken in a normal tone; 4, lethargic response to name being called in a normal tone; 3, responds only after name is called loudly or repeatedly (or both); 2, responds only after name is called loudly and after mild shaking; and 1, does not respond despite name being called and being mildly shaken.

After evaluation of the sedation level, the patients entered the operation room from the day surgery preparation room and lay down on the operating table. Then, a peripheral 22-gauge catheter was inserted into a dorsal hand vein. Patient cooperativeness during transfer and during venous cannulation was closely recorded. The patient’s condition during transfer and when entering the operation room was evaluated by an original score: 1, sleeping; 2, cooperative; 3, fighting. The patient’s condition during venipuncture was also evaluated as: 1, no body movement; 2, cooperative; 3, fighting.

Anesthesia was usually induced with 5 mg/kg of thiamylal sodium (Isozol, Nichi-Iko Pharma Tech Co., Toyama, Japan), followed by 0.6 mg/kg of rocuronium bromide (Eslax, MSD K. K., Tokyo, Japan) to facilitate nasotracheal intubation. After intubation, a nasogastric tube was inserted to withdraw gastric contents. Anesthesia was maintained with 66% nitrous oxide and sevoflurane at an end-tidal concentration of 1 to 2% in oxygen (Fabius Tiro, Dräger Medical Inc., Lübeck, Germany).

When the procedure was expected to be painful, 2% lidocaine with 1:80,000 epinephrine was injected for local anesthesia. When the patient underwent tooth extraction, 1 mg/kg of flurbiprofen axetil (Ropion, Kaken Pharmaceutical Co. Ltd., Tokyo, Japan), up to a dose of 50 mg, was administered intravenously before the end of the operation.

Immediately after the end of the dental procedure, administration of sevoflurane was discontinued. After extubation, agitation score was evaluated according to the scoring system for emergence delirium as (8): 1, sleeping; 2, awake, calm; 3, irritable, crying; 4, inconsolable crying; 5, severe restlessness, disorientation.

Thereafter, patients were shifted from the operating room to the post anesthesia care unit (PACU). Blood pressure, pulse rate, arterial oxygen saturation (SpO2), body temperature, and modified post anesthetic discharge scoring system (mPADSS) scores (9,10) were measured and evaluated every 30 or 60 min until discharge. Recovery time was defined as the time from extubation until the patients obtained a mPADSS score ≥ 9. Actual discharge was permitted when the patients satisfied the mPADSS score ≥ 9 criterion and after confirmation of the ability to drink fluids and adequate urine output while in the PACU.

- Statistical analyses

In this study, the primary predictor variable was the administration method of midazolam. The outcome variables measured were OAA/S Scale score, transfer condition while entering the operation room, venipuncture condition, post-anesthetic agitation and recovery time. The third category of variables studied included gender, age, height, weight, anesthesia time, operation time and coexisting disabilities.

Distributions of patients’ values were tested for normality using the Shapiro-Wilk test. Since distribution of anesthesia time and operation time were non-normal (*P* < 0.05), the Mann-Whitney test was used for analysis. These data are expressed as median (interquartile range). Parameters with normal distributions (*P* > 0.05) and equal variance (*P* > 0.05), such as age, height, body weight and recovery time, were analyzed by the Levene and unpaired t tests. These data are expressed as mean ± standard deviation. Patient gender, medications, coexisting disabilities, OAA/S scale score, transfer condition, venipuncture condition and agitation score were compared by the chi-square test. Statistical analysis was performed with the SPSS software package (SPSS version 22.0; IBM Corp., Armonk, NY, USA). A *P* value less than 0.05 was considered statistically significant.

## Results

**Figure 1:**
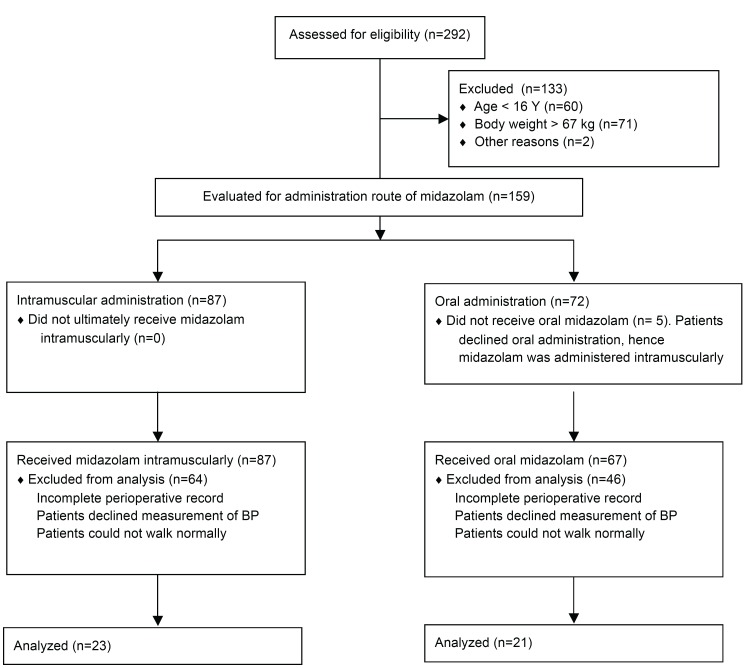
Flow diagram demonstrating patient inclusion in the study.

Anesthesia records of 159 patients were reviewed and a total of 44 patients were finally analyzed. Twenty-three patients were administered midazolam intramuscularly and 21 orally (Fig. 1).  Table 1 presents the clinical characteristics and procedural summary of all patients. There were no significant differences in age, gender, height, body weight, medications such as tranquilizers and anticonvulsant drugs, anesthesia time, operation time and recovery time between the two groups. There were also no significant differences in the number of patients with and without mental retardation, autism, cerebral palsy and epilepsy.

**Table 1:**
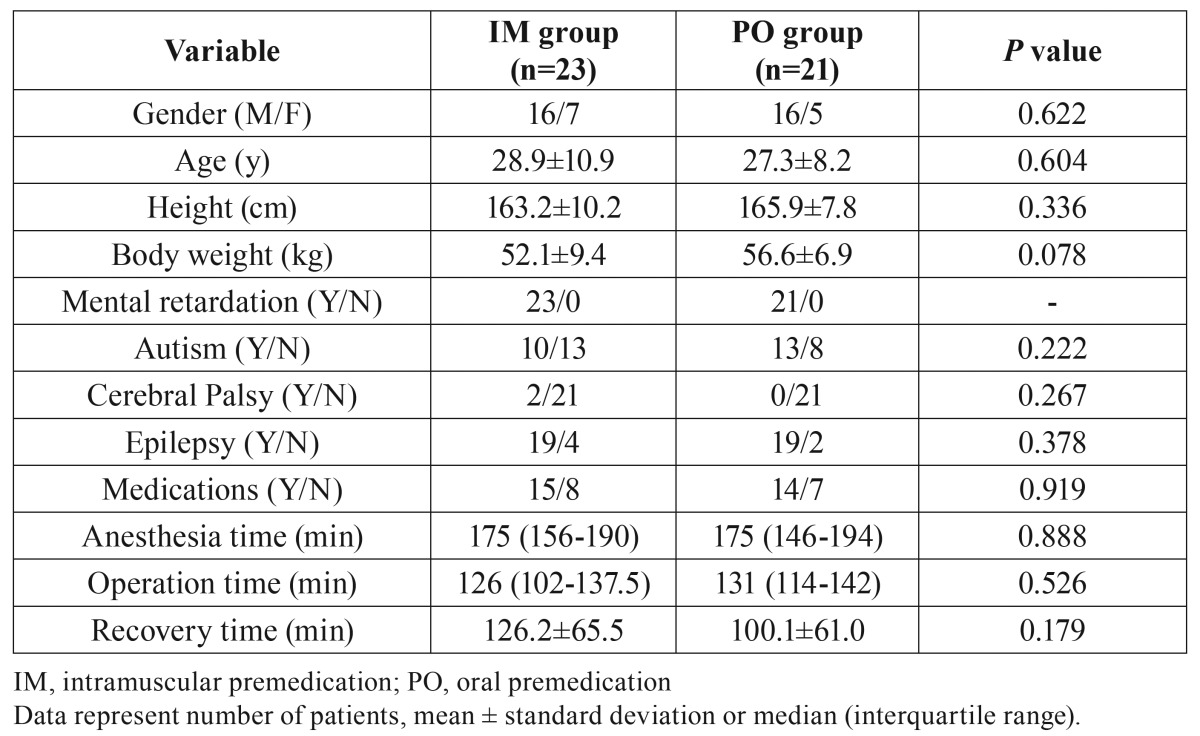
Patients’ clinical characteristics and procedural summary.

The sedation level after premedication is shown in  Table 2. An OAA/S score of 4 was most frequent in both groups, while some patients were deeply sedated. However, there were no significant differences in the sedation level after premedication between the two groups.

**Table 2:**
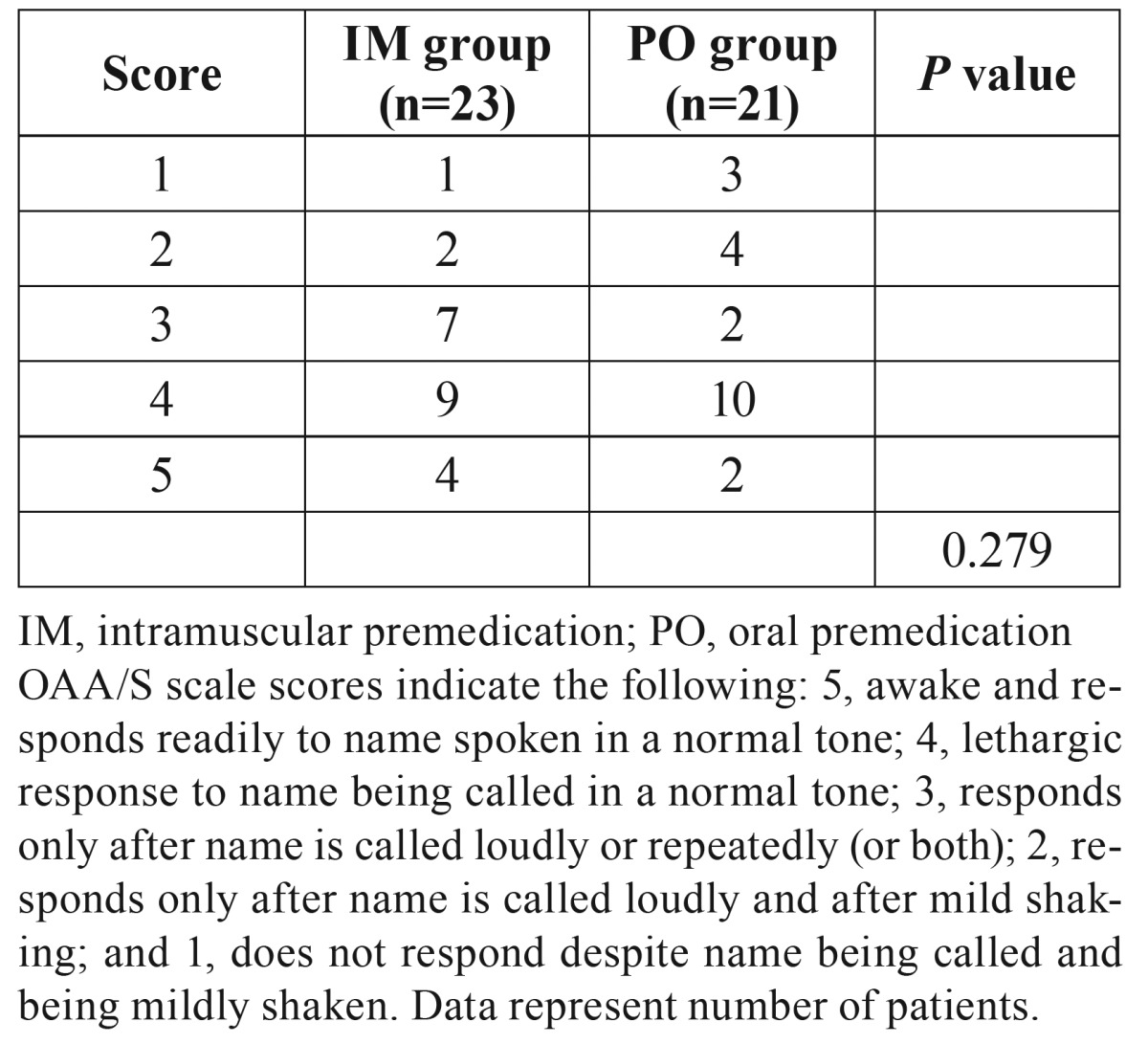
Observer’s Assessment of Alertness/Sedation (OAA/S) scale scores after administration of midazolam.

The patients’ condition at the time of transfer to the operating room is shown in  Table 3. Over 90% of the patients (41/44) were transferred to the operating room without any resistant behaviors. There were no significant differences in transfer condition scores between the two groups. The condition of the patients at the time of intravenous cannulation is also shown in  Table 3. The score of cannulation condition was significantly lower in Group PO than in Group IM (*p*=0.034). More patients in Group PO were more deeply sedated than patients in Group IM during venous cannulation. However, 3 patients exhibited resistant behaviors in each group.

**Table 3:**
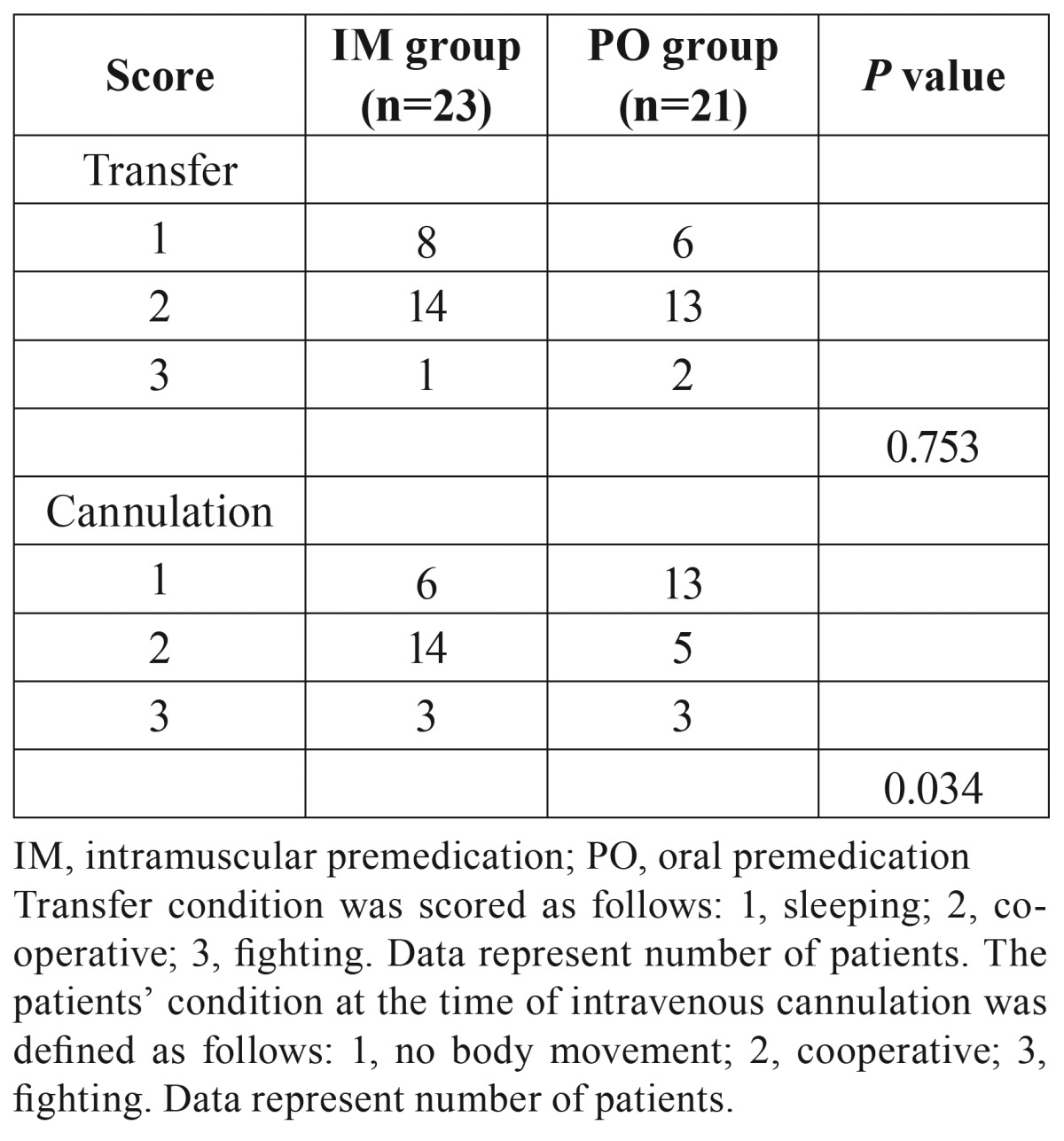
Patients’ condition at the time of transfer in to the operation room and intravenous cannulation.

Agitation scores after extubation are shown in  Table 4. There were no significant differences in the score of transfer condition and agitation after extubation between the two groups.

**Table 4:**
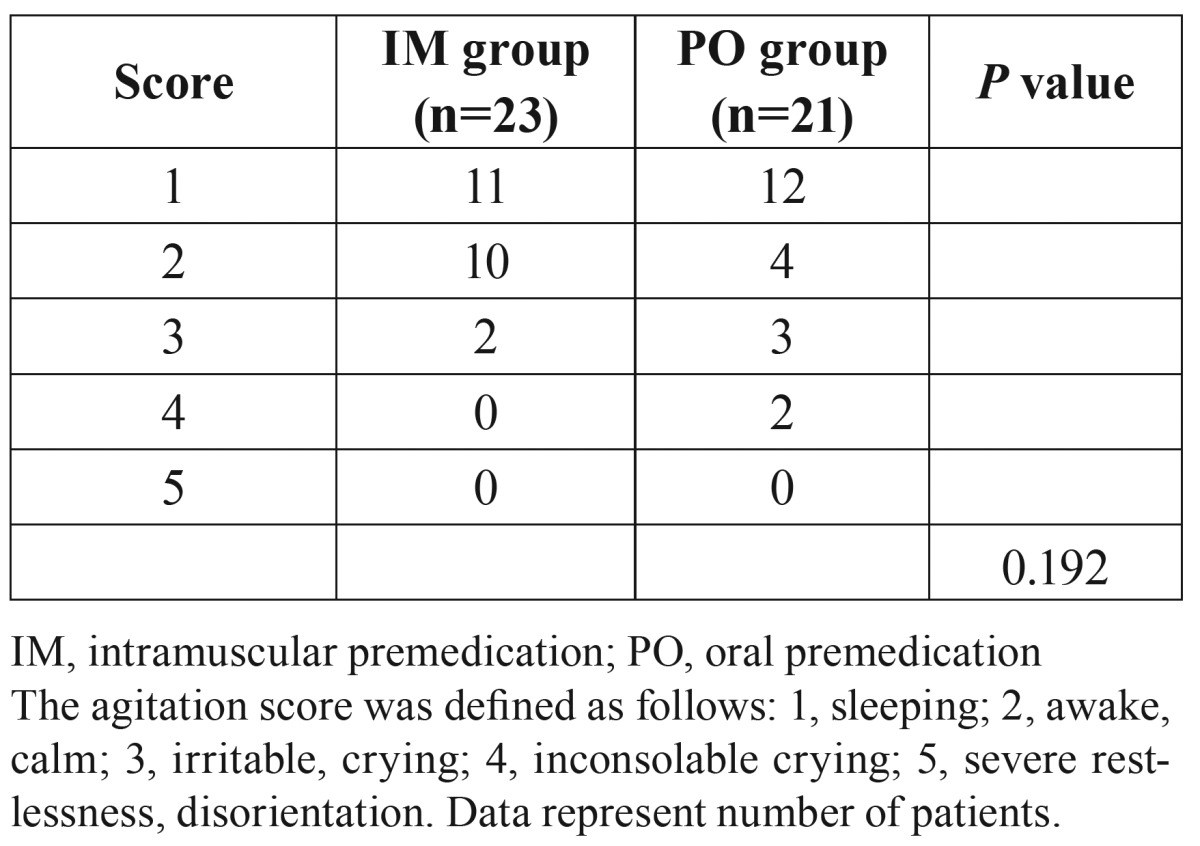
Agitation scores after extubation.

## Discussion

The main finding of this study was that 0.3 mg/kg of oral midazolam was more effective in facilitating intravenous cannulation than 0.15 mg/kg of intramuscular midazolam. Although there were no differences in OAA/S scale scores after sedation and in the post-anesthesia transfer condition between the two groups, 0.3 mg/kg of oral midazolam could reduce venipuncture-related anxiety.

The optimal intramuscular and oral dose of midazolam for patients with intellectual disability is not known. In normal adult patients, the optimal premedication dose of intramuscular midazolam was reported to be 0.08 mg/kg (11). However, based on our clinical experience, we believe that a larger dose is required in patients with intellectual disability. On the other hand, in pediatric patients, 0.5 mg/kg of oral midazolam reportedly provides effective sedation when it is used as premedication before general anesthesia (12). However, it seems inappropriate to use the same dose for uncooperative pediatric patients and for adult patients with intellectual disability. If 0.5 mg/kg of midazolam is given orally to adult patients, it is perfectly possible that side effects such as delayed recovery due to over dosage may occur. This complication should be avoided, especially in ambulatory anesthesia.

There are a few studies on oral midazolam premedication for intellectually disabled patients. Maeda *et al.* used a small dose of oral midazolam (an average of 0.042 mg/kg) before induction of general anesthesia when intravenous cannulation was difficult (13). They reported the usefulness of midazolam premedication to reduce fear. However, the same group also reported that 0.3-0.5 mg/kg of oral midazolam before induction of sedation provided adequate sedation (14). The reason for the difference in the dose of midazolam between general anesthesia and sedation was unmentioned. Collado *et al.* used 0.3 to 0.5 mg/kg of oral midazolam and if necessary, additional inhalation sedation (50% nitrous oxide/oxygen), and demonstrated the improvement in patient cooperation (modified Venham scale 0-3) during venous cannulation in about three-quarters of patients (15). Asahi *et al.* gave 0.3 mg/kg of oral midazolam to combative patients (16).

Therefore, the dose of oral midazolam was determined to be 0.3 mg/kg and a maximum dose of up to 20 mg (6) was used in the present study. The dose of intramuscular midazolam was determined to be 0.15 mg/kg (6,17), because 50% of orally administered midazolam reaches the systemic circulation due to the hepatic first-pass effect (18).

Midazolam premedication has an 86% success rate in terms of improvement in intravenous cannulation conditions (38/44: Cannulation condition score 2 or 3/ Group IM and PO). The proportion of patients with a cannulation score of 3 was 61.9% (13/21) in Group PO and 26.1% (6/23) in Group IM. Our results suggest that 0.3 mg/kg of oral midazolam provides adequate sedation during intravenous cannulation. In the present study, there was no difference in recovery times between the groups. The effect of oral midazolam on recovery time is still controversial.

In pediatric patients, Bevan *et al.* (19) showed prolonged recovery after oral premedication with midazolam 0.5 mg/kg, and Mishra *et al.* (20) reported delayed recovery associated with high dose oral midazolam administration (1.0 mg/kg). However, Horgesheimer *et al.* reported that 0.5 mg/kg of oral midazolam premedication did not delay discharge of children undergoing general anesthesia for dental treatment (21). The systematic review from Cox *et al.* also demonstrated that premedication with oral midazolam 0.5 mg/kg has minimal effect on recovery time (12).

Maeda *et al.* demonstrated that oral midazolam was associated with delayed recovery after dental sedation (14) and ambulatory general anesthesia (13). However, their mean treatment time was shorter (43.1 min under sedation and 88.0 min under general anesthesia) and prolonged treatment time (>100 min under general anesthesia) did not contribute to delayed recovery. In the present study, mean operation time was so long (131 min in Group PO) that oral midazolam probably did not affect recovery time.

As an administration route for midazolam, the oral route is more comfortable than other routes, such as intramuscular, intranasal and intrarectal routes. Some investigators compared the administration routes of midazolam. One study compared intramuscular and intranasal administration of midazolam (22). The intramuscular route allowed for a better sedation level and less movement at the time of venous cannulation than the intranasal route. Malinovsky *et al.* (23) compared 0.2 mg/kg intranasal, 0.3 mg/kg rectal and 0.5 mg/kg oral administration of midazolam and concluded that the intranasal route had the most rapid onset of sedation. However, application of the intranasal administration route to patients with intellectual disability is difficult because of their uncooperative and combative attitude.

Although our results showed the superiority of 0.3 mg of oral midazolam, oral administration has two disadvantages. First, onset time through the oral route is longer than that with the intramuscular route. It takes about 30 min to achieve the maximum sedative effect based on the peak effect (6,17). Second, patients with intellectual disability do not always accept orally administered medicine. Some patients may refuse to drink a drug solution or spit it out after taking it into their mouth. In our hospital, midazolam is mixed with a sweetened syrup (24) to make it more palatable. If a patient refuses to drink the midazolam solution, the drug is administered intramuscularly.

There are some limitations to the present study. First, most patients were on regular medication, such as anticonvulsant and anxiolytic drugs, which might affect the effectiveness of midazolam. However, it was neither possible to omit these drugs before anesthesia, nor to implement a study using these drugs, because of the variety of drugs and doses received by the patients. Second, the main biases of our study are that it was a retrospective, uncontrolled and unblinded study with a small sample size. We could not implement a randomized controlled prospective protocol for this study because many patients’ parents or caregivers requested the oral route of midazolam administration when providing informed consent, and hence, we could not randomly allocate patients to the two groups. The sample size of this study was small because many patients were excluded due to insufficient information on outcome variables. Moreover, the administration route was not blinded to the evaluator. Therefore, the possibility of selection and observer bias exists in the present study. Third, the study patients had different pathologies and degrees of intellectual disability. Asahi *et al.* (16) demonstrated that autistic patients require more propofol compared with intellectually impaired patients during dental treatment under intravenous general anesthesia. Since the difference in the pathology and degree of intellectual disability was not considered in the present study, further study on the influence of these different pathologies on the required dose of midazolam should be explored. Fourth, because the oral dose of midazolam was limited to a maximum of 20 mg, patients weighing over 67 kg were excluded from the study. Therefore, we cannot provide useful information about the appropriate midazolam dose in obese patients.

## Conclusions

As premedication for intellectually disabled dental patients, 0.3 mg/kg of oral midazolam was more effective than 0.15 mg/kg of intramuscular midazolam in terms of patient cooperation for venous cannulation, without resultant prolongation of recovery time. If both oral and intramuscular midazolam are acceptable, oral administration is recommended due to less resistant behaviors by the patient for venous cannulation.
